# Evaluation of the Superiority of Lightweight-Aggregate-Concrete Prestressed Box Girders in Terms of Durability and Prestress Loss

**DOI:** 10.3390/ma16196360

**Published:** 2023-09-22

**Authors:** How-Ji Chen, Cheng-Chang Kuo, Chao-Wei Tang

**Affiliations:** 1 Department of Civil Engineering, National Chung-Hsing University, 145 Xingda Road, South District, Taichung City 40227, Taiwan; hojichen@dragon.nchu.edu.tw (H.-J.C.); Cchkuo@freewaygov.tw (C.-C.K.); 2 Department of Civil Engineering and Geomatics, Cheng Shiu University, No. 840 Chengching Road, Niaosong District, Kaohsiung 83347, Taiwan; 3 Center for Environmental Toxin and Emerging-Contaminant Research, Cheng Shiu University, No. 840 Chengching Road, Niaosong District, Kaohsiung 83347, Taiwan; 4 Super Micro Mass Research and Technology Center, Cheng Shiu University, No. 840 Chengching Road, Niaosong District, Kaohsiung 83347, Taiwan

**Keywords:** mechanical properties, time-dependent deformation, creep, shrinkage, chloride ion

## Abstract

This case study aimed to compare the differences in the durability and prestress loss between normal-weight-concrete (NC) and lightweight-aggregate-concrete (LWC) prestressed box girders, which were constructed at the same time in the same area, so as to verify the superiority of using synthetic lightweight aggregate (LWA) made from reservoir sediments in prestressed bridges. For the NCs and LWCs used in the prestressed box girders, the basic mechanical properties (compressive strength, flexural strength, splitting tensile strength, and elastic modulus) were tested, as well as the durability properties (chloride ion penetration resistance and rapid chloride permeability). Then, through the prestress-monitoring system, the prestress losses of the two groups of prestressed box girders were tracked. The results of the durability test confirmed that LWC can inhibit the penetration of air, water, and chloride ions by strengthening the interfacial transition zone between the aggregate and the cement paste, thereby improving its durability. Moreover, the magnetic-flux prestress loss of the NC prestressed box girder reached 8.1%. In contrast, the magnetic-flux prestress losses on both sides of the LWC prestressed box girder were 4.6% and 4.9%, respectively. This verified that, under the same environmental conditions, the use of LWC produced less of a prestress loss than the use of NC.

## 1. Introduction

Lightweight aggregate (LWA) is a general term for natural or synthetic aggregates with weights from 80 to 900 kg/m^3^ [1]. Lightweight-aggregate concrete (LWC) can be produced by using LWAs instead of normal-weight aggregates [2]. Since S. J. Hayde invented the technology of producing expanded-clay LWAs in a rotary kiln in 1917, the progress of synthetic LWA technology has promoted the improvement in the quality of LWAs, thereby accelerating the development of high-strength LWC technology [3]. In the past two decades, in order to reduce the consumption of natural resources, the source of materials for the production of synthetic LWAs has developed toward resource recycling [4]. For example, industrial waste and municipal solid waste are used as renewable resources to produce LWAs [5,6,7,8,9,10,11,12,13,14,15]. Generally speaking, the shell layer of synthetic high-strength LWAs and high-performance LWAs is hard and dense, but the interior is porous; thus, it is light in weight and has appropriate strength. Therefore, LWCs that are mixed with these excellent synthetic LWAs have the advantages of being lightweight as well as having heat insulation, fire resistance, seismic resistance, and high-strength properties [16]. The unit weight of LWCs is greatly affected by the type of LWA, the composition of materials, and the environmental conditions of conservation. For the maximum limit of the LWC unit weight, the relevant standards of various countries have clear regulations according to their respective resource conditions and technical requirements. For example, according to ACI 213R-14 [17], structural LWC is defined as having a 28-day compressive strength exceeding 17 MPa and a 28-day air-dried unit weight not exceeding 1850 kg/m^3^.

Expanded-shale, -clay, and -slate LWAs are highly absorbent, yet they are composed of vitrified silicates and are particularly durable [18]. In addition, LWAs have other unique properties that can improve the durability of LWCs, such as the elastic compatibility between LWAs and cement paste, the ability of LWAs to promote internal curing, and the lower rigidity of LWAs [18]. In particular, a relatively dense interfacial transition zone (ITZ) is formed between LWAs and cement paste, which results in LWCs having good durability. Many studies have shown that the ITZ of LWCs is much better than that of NCs, which is due to the improved adhesion between LWAs and cement paste [19,20,21,22,23,24,25,26,27]. Moreover, structural LWCs can provide a more effective strength ratio (ratio of strength to weight) than normal-weight concretes (NCs). Therefore, structural LWCs with unit weights of 1400–2200 kg/m^3^ have been widely used in various structural projects, such as high-rise buildings, bridges, prestressed members, and offshore oil platforms, and their use in these contexts has shown good development and prospects [17,28]. However, the basic components of LWCs, their interactions, and their effects on the mechanical properties and durability are significantly different from those of NCs [29]. This difference is attributed to the composition of the mortar matrix and the LWAs used [25,30]. In NCs, the elastic modulus and strength of normal-weight aggregates are greater than those of the mortar, and the aggregate is the main load-bearing system. Once the applied stress exceeds the tensile strength of the mortar, the mortar will crack first and penetrate the entire mortar [31,32]. In contrast, when LWC is stressed, the situation is more complicated, as it depends on the elastic modulus of the LWAs and whether its strength is higher or lower than that of the mortar [33]. Therefore, the complex relationship between the two materials makes the mechanical behavior and collapse mechanism of LWCs applied to reinforced-concrete and prestressed-concrete members quite different from those of NCs [34,35]. In general, the elastic modulus of LWCs may be 15–60% lower than that of NCs of the same strength class, depending on the density of the concrete and the aggregate used [36,37]. In view of this, certain design codes have put forward specific suggestions for structural LWC, as well as have evaluated reinforced-LWC members by means of their strength reduction coefficients [38]. Most of the reduction values are based on the experimental results of traditional LWC members. For example, the ACI 318 building code [39] uses a correction factor based on the density of the concrete to account for the elastic modulus, tensile strength, shear strength, and torsional strength of LWCs in comparison to NCs with the same compressive strength.

Many studies have shown that LWCs perform much better than NCs under variable and dynamic loads [40,41]. In particular, the lighter structure of LWCs ensures higher natural frequencies, lower vibration amplitudes, and higher damping. Therefore, the application of LWCs in bridge engineering has been quite common [42]. For prestressed concrete structures, information on the actual state of the prestress is an important basis for determining their load-carrying capacities and remaining service lives [43]. Prestress loss refers to the slow reduction in the induced compressive stress in a prestressed part due to various factors. There are two main types of prestress loss. The first type is the instantaneous elastic-shortening loss; the second type is the long-term loss, which is mainly caused by the relaxation of the prestressing strands that results in the creep and shrinkage of the concrete [44,45]. Prestress loss is also affected by other time-dependent concrete properties, such as the compressive strength and the modulus of elasticity. Bymaster et al. [46] advised that LWCs have large prestress losses. This is due to the lower elastic modulus of LWAs with lower stiffness, thereby leading to the expected greater elastic shortening of LWC members. The study by Chen et al. [16] showed that, after 180 days of prestressing, the prestress loss of full-scale self-consolidating lightweight-aggregate-concrete members was around 5.35–6.83%, which was lower than that of conventional self-consolidating-concrete members (approximately 8.19–9.06%). Kraľovanec et al. [43] concluded that the prestress loss of the prestressed components is affected by the construction stage, the materials used, the prestressing technology, or the required service life of the component.

The creep control and shrinkage control of concrete are other important factors that affect the performance of prestressed concrete members [31,32,47,48,49]. Most researchers recognize that shrinkage and creep are always higher for LWCs than for NCs [48,50,51,52]. This is because the stiffness (that is, the elastic modulus) of LWAs itself is lower than that of ordinary aggregates, which makes the restraining effect of aggregates on the autogenous shrinkage of cementitious materials weaker, thereby resulting in larger drying shrinkages and degrees of creep in LWCs than in NCs. Report BE 96-3942/R2 [53] stated that the creep strain of LWCs is maybe 20–60% higher compared to concrete with normal-weight aggregates. However, the results of Nilsen and Aitcin [54] showed that the drying shrinkages of LWCs made of expanded shale were from 30% to 50% lower than those of NCs. Furthermore, Lopez et al. [55] showed that high-performance LWCs mixed with expanded-slate LWAs exhibited less creep and slightly greater shrinkage than the general HPC of a similar paste, mix design, and strength. Rodacka et al. [56] showed that the final value of the test for the shrinkage deformation of LWCs was 38% lower than the value estimated according to Eurocode EN-1992-1-1 [57]. Furthermore, the final creep deformation of the tested LWCs was more than two times lower than that of the corresponding NCs. The study by Szydłowski and Łabuzek [34] showed that compared with NCs, LWCs with higher strength, especially high-strength LWCs, can exhibit similar—and sometimes even lower—creep strains [58,59,60]. Kayali [60] showed that different types of LWAs produce distinct drying shrinkage behaviors.

**Figure 1:**
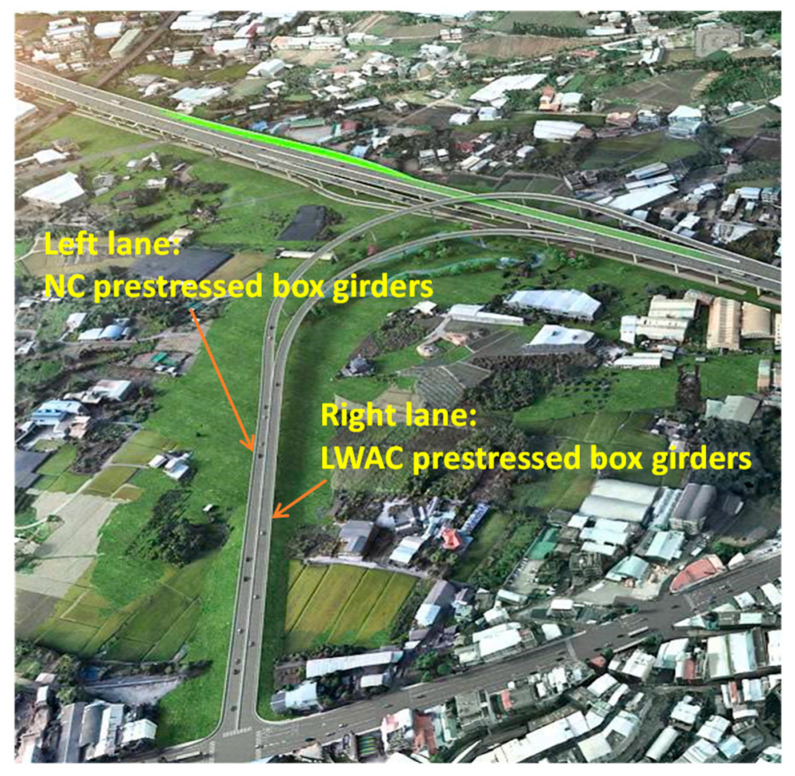
Schematic diagram of the location of the NC and LWC prestressed box girders.

**Figure 2:**
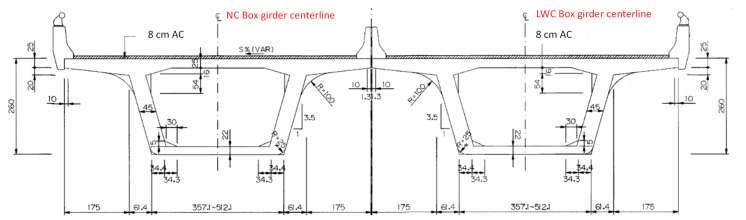
Standard cross section of the NC and LWC prestressed box girders.

**Figure 3:**
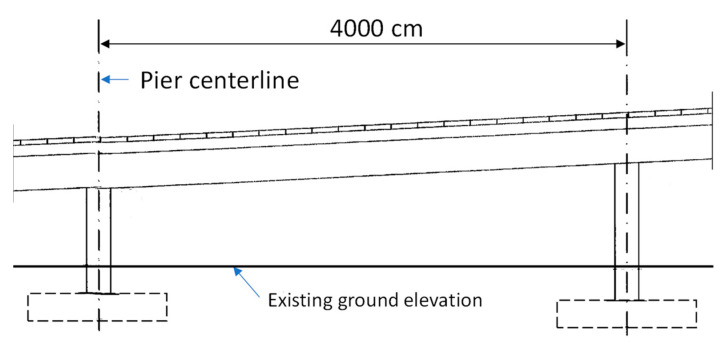
Standard longitudinal section of the NC and LWC prestressed box girders.

The surface of Taiwan is covered with a large amount of shale and slate, which is washed into rivers, turned into sediments, and accumulated in reservoirs, forming an excellent source of LWAs. In the past two decades, Taiwan has successfully sintered LWAs with excellent properties from reservoir sediment and applied them to structural engineering [7,8,9]. In view of the full application of LWC in bridge engineering in European and American countries, Taiwan’s Ministry of Transportation is actively promoting LWC in road bridges. In order to verify the superiority of applying synthetic LWAs from reservoir sediments to prestressed bridges, the Taiwan Highway Bureau selected an interchange bridge located in central Taiwan (as shown in Figure 1), and this was achieved using concrete poured with synthetic LWAs as the structural material. In view of the importance of evaluating the prestress loss and durability in prestressed concrete structures, a monitoring plan for an LWC prestressed box bridge with a span of 40 m in the viaduct was implemented. Another NC prestressed box bridge with a similar structural cross section was selected from another adjacent bridge section (as shown in Figure 1), and the same monitoring and testing operations were carried out as a control group. The standard cross section and longitudinal section of the NC and LWC prestressed box girders are shown in Figure 2 and Figure 3, respectively. This research team was commissioned by the Taiwan Highway Bureau to conduct this project study. In this case study, according to the construction progress of the bridge, magnetic-flux-monitoring instruments were installed on site to monitor the prestress changes in the prestressed steel tendons in the bridge components over a long period of time. Moreover, the time-dependent-deformation and durability test results of the concrete at the construction site and in the laboratory were analyzed. The monitoring and testing of the related items of this case study included various property tests of the concrete (basic performance, time-dependent deformation, and durability) and the monitoring of the prestress loss of the prestressed steel tendons. The results obtained from this actual monitoring and testing can be used as a reference for Taiwan to promote the design of lightweight-aggregate-concrete prestressed bridges in the future.

## 2. Materials and Methods

### 2.1. Materials

**Figure 4:**
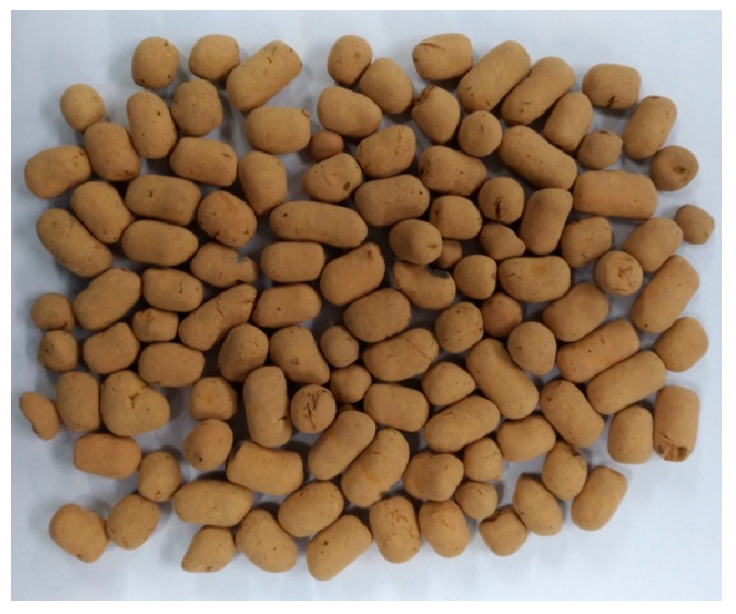
Appearance of synthetic LWAs that were created using reservoir sediments.

The materials used in this case study and their sources are described below:

- Cement: a locally produced Type I Portland cement, with a specific gravity of 3.15 and a fineness of 3550 cm^2^/g; its chemical composition is shown in Table 1;
- Slag: purchased from Yu Qingtang Enterprise, Taichung, Taiwan; its specific gravity was 2.89, and its chemical composition is shown in Table 1;
- Fly ash: taken from Taichung Thermal Power Plant, Taichung, Taiwan; its specific gravity was 2.32, and its chemical composition is shown in Table 1;
- Silica fume: purchased from Elkem Taiwan, Taichung, Taiwan; its specific gravity was 2.1, and its chemical composition is shown in Table 1;
- Water: general tap water, which was in line with the general quality requirements of concrete mixing water;
- Fine aggregate: a natural river sand with an FM value of 2.67, a specific gravity of 2.63, and a 24 h water absorption rate of 1.3%;
- Coarse aggregate: a natural crushed stone with a specific gravity of 2.64 and a 24 h water absorption rate of 0.7%;
- Lightweight aggregate: the appearance of the synthetic LWA obtained by using reservoir sediments used in this case study is shown in Figure 4, and its basic properties are shown in Table 2;
- Superplasticizer: purchased from An-Yao Company; it met the requirements of ASTM C494-81 [61] Type F.

### 2.2. Mix Proportions of Concrete

In this case study, the specimens of the control group (NC) and the experimental group (LWC) were made. Based on the design load of the prestressed box girders, the target values of the 28-day compressive strengths of the NC and LWC were 50 and 40 MPa, respectively. To ensure the strength and durability of the LWC, supplementary cementitious materials were incorporated, and a lower water-to-cement ratio was used. Considering the workability, strength, and durability of concrete, the amount of each material of the two groups of concrete was determined via trial mixing. The NC was made with a water–binder ratio of 0.36, a cement content of 364 kg/m^3^, and a slag content of 90 kg/m^3^, while the LWC was made with a water–binder ratio of 0.39, a cement content of 296 kg/m^3^, and a supplementary cementitious material content of 243 kg/m^3^. The mix proportions of the two groups of concrete are shown in Table 3.

### 2.3. Casting and Curing of Specimens and Test Methods

Before mixing the LWC, the LWAs were pre-wetted and soaked in a water tank for 24 h. During the pouring operation of the bridge, the sampling of the two groups of concrete and the preparation of the specimens were carried out simultaneously for subsequent concrete property tests. For each test age, three specimens were produced for each test. Air-dry unit weight and compressive-strength tests shared the same specimen. Three test specimens were produced for each age of compressive strength, with a total of 72 test specimens for the two groups of concrete. Three test specimens were made for each age of flexible strength, with a total of 24 test specimens for the two groups of concrete. Splitting tensile strength only produced three specimens for one test age, and the two groups of concrete had a total of six specimens. The elastic-modulus and compressive-strength tests shared the same specimen. Three specimens were produced for the drying shrinkage test, and there were six specimens in total for the two groups of concrete. In the creep test, three specimens were produced in each group, with a total of 18 concrete specimens in the two groups. The test items and specimen ages, as well as the test specifications of the basic properties and time-dependent deformation of the concrete, are shown in Table 4. After pouring the specimens on site, the specimens were divided into two parts. One was for on-site curing, which kept the specimens moist with mist spray for 7 days. After that, the specimens were subjected to air curing. The other part was for laboratory standard curing. The temperature in the curing room was controlled at 23 ± 2 °C, and the relative humidity was greater than 95%. The specimens were taken out one day before the planned curing age for various basic-property tests.

**Figure 5:**
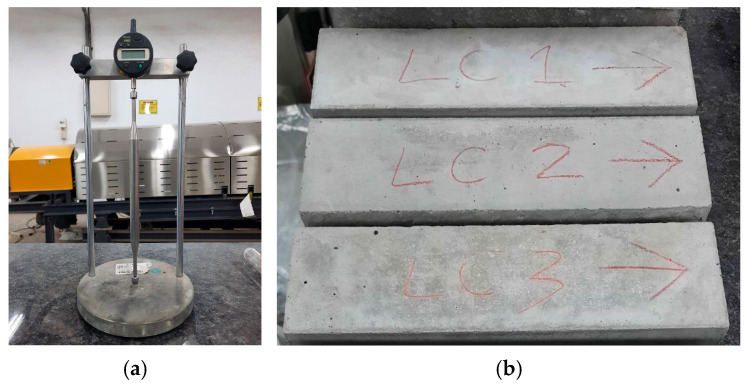
Drying shrinkage test: (**a**) equipment and (**b**) specimens. LC1–LC3 are the specimen numbers. The arrows are used to indicate the direction in which the specimen is placed on the instrument.

The air-dry unit weight of concrete cylinders (15 cm × 30 cm) at the age of 90 days was measured according to the ASTM C567 [67]. The concrete specimens were tested for compressive strength (ASTM C39 [68]), flexural strength (ASTM C78 [69]), splitting tensile strength (ASTM C496 [70]), elastic modulus (ASTM C469 [71]), drying shrinkage (ASTM C157 [72]), and creep strain (ASTM C512 [73]). The specimens of the drying shrinkage test adopted the on-site-curing method, and the test equipment and specimens are shown in Figure 5. In addition, the specimens of the creep test also adopted the on-site-curing method.

In essence, concrete is very chemically stable and has excellent durability. In view of this, when evaluating the durability of reinforced-concrete structures, the evaluation is mainly based on whether the steel bars are corroded and thereby damage the structure [17]. Regarding the durability evaluation tests of reinforced-concrete structures, the compactness of the concrete is used as the research factor. In other words, the better the concrete’s ability to resist the intrusion of external substances, the higher the durability of the reinforced-concrete structure. According to the discussion content and test methods of the durability of LWC in the ACI 213R-14 [17], this case study planned durability-related tests. Among them, the chloride ion penetration resistance test (CNS 14795 [74]) is a passive chloride ion penetration test based on the amount of charge. Although the test method is simple and fast (6 h), the reference value in research is low. Therefore, the immersion test (ASTM C1543 [75] or CNS 15649 [76]) is added to test the penetration of chloride ions into concrete in its natural state, and the test results are more convincing. In terms of the concrete durability tests, the test items, specimen ages, and test specifications of the concrete durability are shown in Table 5. There were three test ages for each group of concrete, and three specimens were poured for each age. The two groups of concrete had a total of 72 specimens. After 24 h of casting, the specimens were de-molded. Afterward, the specimens were subjected to laboratory standard curing until they were taken out one day before the planned age. Thereafter, various durability tests were performed.

**Figure 6:**
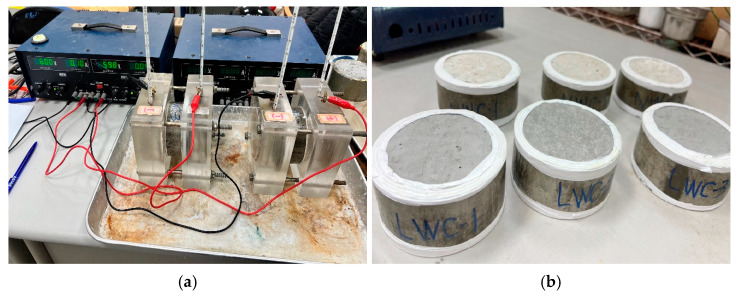
Rapid chloride permeability test: (**a**) equipment and (**b**) specimens.

The ultrasonic test was carried out with reference to ASTM C597 [77]. The ultrasonic wave was transmitted through the instrument to penetrate the concrete specimen to detect its wave velocity, thereby estimating the compactness of the concrete. The chloride ion penetration resistance test was carried out with reference to CNS 14795 [74]. During the 6 h test period, the current passing through a concrete sample with a thickness of 5 cm and a nominal diameter of 10 cm was measured (as shown in Figure 6). After calculating the total passing electricity, the current was expressed in coulombs to evaluate the ability of the concrete to resist the penetration of chloride ions.

**Figure 7:**
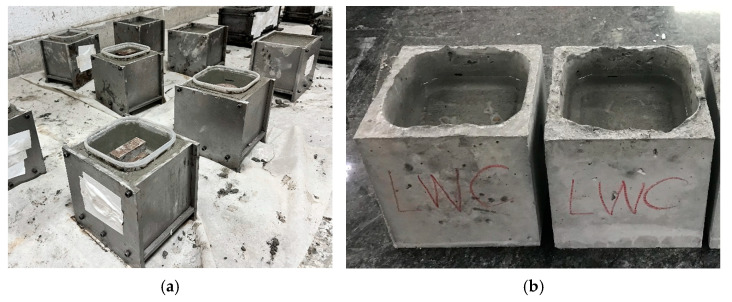
Chloride ion penetration test: (**a**) situation following specimen pouring and (**b**) the specimens containing sodium chloride solution.

The chloride ion penetration test was carried out according to ASTM C1543 [75] and CNS 15649 [76]. The size of the specimen was 15 cm × 15 cm × 15 cm, and its center was hollowed out, as shown in Figure 7. A 3% sodium chloride solution was poured into the center of the concrete specimen. After the concrete was soaked, the sample slices were taken out according to the required depth and ground. According to CNS 14702 [78], the content of the chloride ions was measured. Among them, the specimens not immersed in the solution were used for comparison. In addition, the crumble at the junction of the aggregate and the cement paste in the center of the cylinder after the compression test was taken as a sample for microscopic observation with an electron microscope.

### 2.4. Prestress-Loss Monitoring of Prestressed Tendons

**Figure 8:**
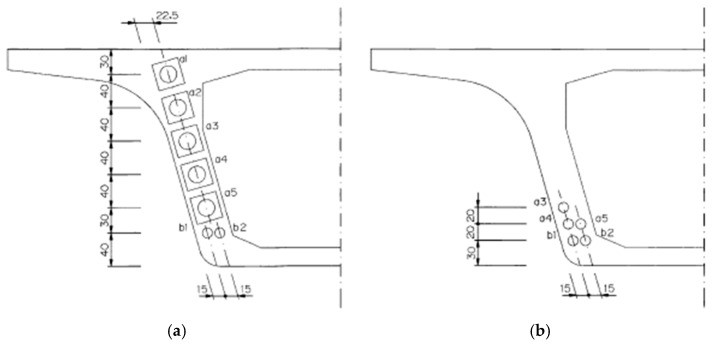
The configuration of prestressed tendons: (**a**) support end and (**b**) span center. a1–a5 and b1, b2 in the figure are the sleeve numbers of the prestressed tendons.

**Figure 9:**
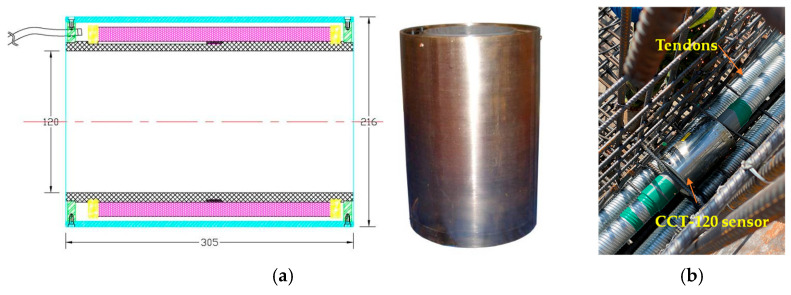
Magnetic-flux cable force measurement system: (**a**) CCT-120 sensor and (**b**) configuration.

The configuration of the prestressed tendons of the NC and LWC prestressed box girders is shown in Figure 8. In this case study, a magnetic-flux cable force measurement system was used to monitor the prestress loss of the tendons of the prestressed-concrete box girders. Using a magnetic-flux sensor to measure the tendon preload is a new method that has been tried in recent years. The measurement principle is based on the fact that the stress on steel is the main sensitive factor that directly affects its magnetic permeability. Therefore, the pre-force value can be calculated by measuring the change in the magnetic permeability of the tendon. When the magnetoelasticity instrument applies a pulse voltage signal to the exciting coil, the exciting coil will generate a magnetic field in the tendon, and—at the same time—an induced voltage will be generated in the measuring coil. When the tendon is changed by the stress of the load, the magnetic-field strength inside the tendon will also change, and—at the same time—the induced voltage in the measuring coil will also change. Therefore, the magnetoelastic instrument detects the slight change in the induced voltage on the measuring coil, calculates the force of the tendon, and then displays it on the instrument. The installation diagram of the magnetic-flux CCT-120 sensor used in this case study is shown in Figure 9.

## 3. Results and Discussion

### 3.1. Test Results of the Basic Properties and Time-Dependent Deformation of Concrete

#### 3.1.1. Results of the Air-Dried Unit Weight and Compressive-Strength Tests

The test results of the air-dried unit weights of the two groups of concrete are shown in Table 6. Under the standard-curing and on-site-curing modes, the air-dried unit weights of the NCs were 2391 and 2333 kg/m^3^, respectively, while those of the LWC were 1817 and 1820 kg/m^3^, respectively. This shows that the unit weight of the LWCs was only about 76–78% of the NCs, which meant that replacing NC with LWC could reduce the weight of the whole structure by more than 20%, thereby achieving the purpose of reducing the inertial force during earthquakes. In addition, according to ACI 213R-14 [17], the test results of this case study showed that the air-dried unit weight did not exceed 1850 kg/m^3^.

**Figure 10:**
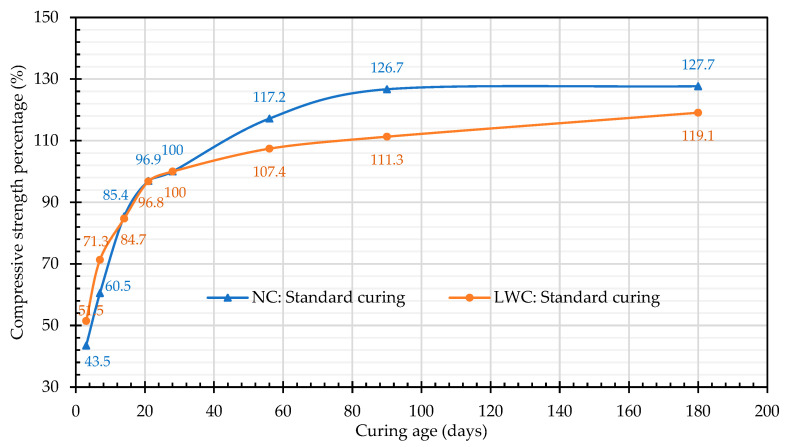
Compressive-strength percentages under standard curing.

The compressive-strength test results are shown in Table 7. The 28-day compressive strengths of both groups of concrete exceeded the target values. It can be seen from Table 7 that the 28-day compressive strengths of the LWCs in this case study exceeded 17 MPa, which met the requirements of ACI 213R-14 [17]. Moreover, it can be seen from Table 7 that the strengths of the two groups of concrete increased with age, especially under standard curing. Under standard curing, the improvement in the concrete strength was particularly obvious. This is consistent with the research results of Wang et al. [79]. This is due to the standard-curing process, which prevents or replenishes the loss of moisture in the concrete while maintaining a temperature that is conducive to hydration. It is worth noting that under standard curing, compared with the 28-day compressive strength, the strength percentages of the LWC at three and seven days of early age were 51.5% and 71.3%, respectively, while those of the NC were 43.5% and 60.5%, respectively, as shown in Figure 10. This shows that the LWC exhibited a higher early-age-strength phenomenon. However, under the two different curing modes, the increase in the compressive strength of the LWC after fourteen days of age was smaller than that of the NC. Under standard curing, the 28-day compressive strength of the LWC was 47.1 MPa, and the 90-day compressive strength was 52.4 MPa. In two months, the strength increased by only 11.3%, which was significantly less than the 26.7% increase in the NC. This is consistent with the research results of Al-Khaiat and Haque [80]: LWC grows faster than NC in terms of early-age strength. In addition, there was a relative increase in the early-age strength of the field-cured specimens. However, the long-term exposure to outdoor sunlight and poor curing conditions caused the strength growth of the specimens to slow down in the later stage. In contrast, the specimens treated with standard curing had a more-than-enough hydration reaction, making the strengths of longer ages higher than those of the on-site curing. Research by Smadi and Migdady [81] showed that the 28-day compressive strength of LWC using expanded-shale LWA was 47–86 MPa and the density was 1720–1940 kg/m^3^. Wu et al. [82] used tuff LWA to produce high-strength LWC with a strength of 55–60 MPa and a unit weight of 1880 kg/m^3^. Accordingly, the compressive strength and density of the LWC in this study should be appropriate.

#### 3.1.2. Results of the Flexural-Strength and Splitting-Tensile-Strength Tests

**Figure 11:**
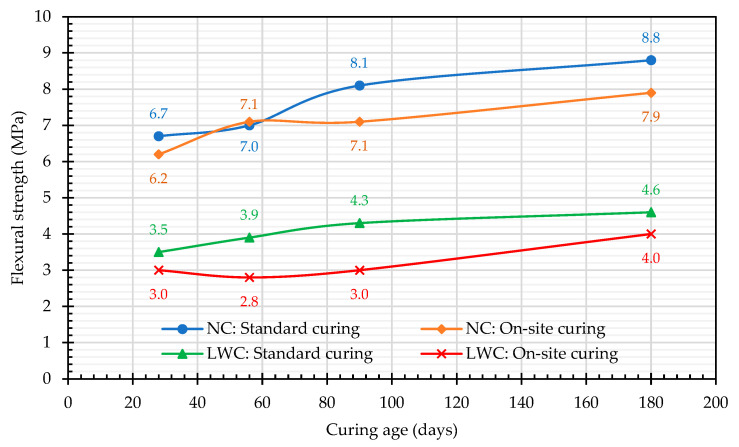
Relationship between the flexural strength and curing age.

The test results of the flexural strength are shown in Figure 11. All fracture surfaces were located within 1/3 of the span of the center of the specimen, indicating that the mixing and pouring of the specimen were quite uniform. The flexural strength of the LWC calculated according to the ACI 318-suggested formula was 3.6 MPa, while the flexural strength of the LWC obtained in this case study was between 3.5 and 4.6 MPa. It can be seen from Figure 11 that the environmental conditions of the on-site curing were affected by weather and were difficult to control, resulting in greater variability in the flexural-strength results of the two groups of concrete specimens. The trends are not really credible due to the high degree of dispersion, resulting in an inability to determine the increase/decrease in the flexural-strength values. In comparison, under standard-curing conditions, the temperature and humidity were easier to control, and the flexible-strength results of the two groups of concrete specimens increased with age. The 28-day flexural strength of the LWC was 3.5 MPa, and the 90-day flexural strength was 4.3 MPa. In two months, the strength increased by 22.9%, slightly higher than the 20.9% of the NC. In addition, regardless of the age, the flexural strength of the standard curing of the two groups of concrete was greater than that of the on-site curing. At the age of 28 days, the flexural strength of the LWC with standard curing was 3.5 MPa, and that with on-site curing was 3.0 MPa. Compared with the compressive strength, it was about 6–7% of the compressive strength. At the age of 28 days, the flexural strength of the NC with standard curing was 6.7 MPa, and that with on-site curing was 6.2 MPa. Compared with the compressive strength, it was about 10–11% of the compressive strength. The results showed that the NC was superior to the LWC in flexural strength.

The test results of the splitting tensile strength are shown in Table 8. At the age of 28 days, the splitting tensile strength of the LWC with standard curing was 3.0 MPa, and that with on-site curing was 2.3 MPa, which was about 4–7% of the compressive strength. At the age of 28 days, the flexural tensile strength of the NC with standard curing was 4.6 MPa, and that with on-site curing was 4.5 MPa. Compared with the LWC, it was about 7–8% with respect to the compressive strength. It can be seen that the splitting tensile strength of the NC was relatively excellent, which was in line with the recommendation of ACI 213R-14 [17].

#### 3.1.3. Results of Elastic-Modulus Test

**Figure 12:**
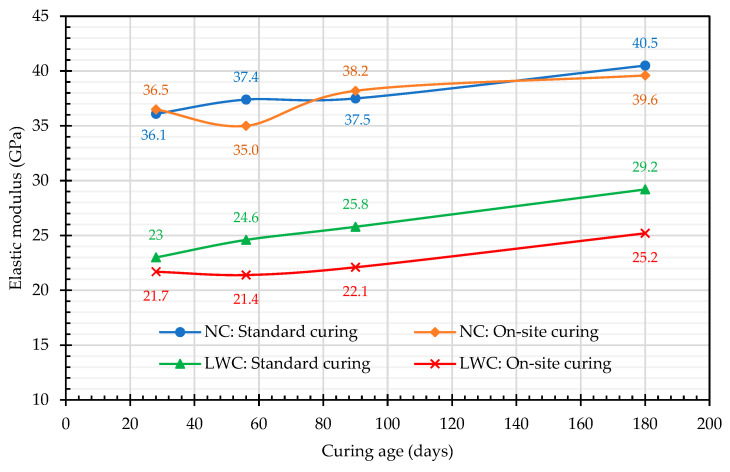
Relationship between the elastic modulus and curing age.

The elastic modulus is an important property of structural LWC in its application. The elastic modulus of LWC mainly depends on its density and strength [83]. The test results of the elastic modulus are shown in Figure 12. Under two different curing modes, the elastic moduli of the LWC and NC at different curing ages ranged from 21.4 to 29.2 GPa and from 36.1 to 40.5 GPa, respectively. These values are consistent with the experimental results reported in the literature [35,84]. For example, typical expanded-clay LWA has an elastic modulus in the range of 10–20 GPa, while ordinary aggregates range from about 30 to 100 GPa—this is the most important difference between the LWCs and NCs used in prestressed members [35]. In addition, the moduli of elasticity of the two groups of concrete increased with age, as shown in Figure 12. The improvement under standard curing was particularly evident, and the improvement rate of the LWC was relatively high. Overall, the elastic modulus of the LWC was about 60–70% of that of the NC. This is consistent with the literature [36,37].

#### 3.1.4. Results of the Drying Shrinkage Test

**Figure 13:**
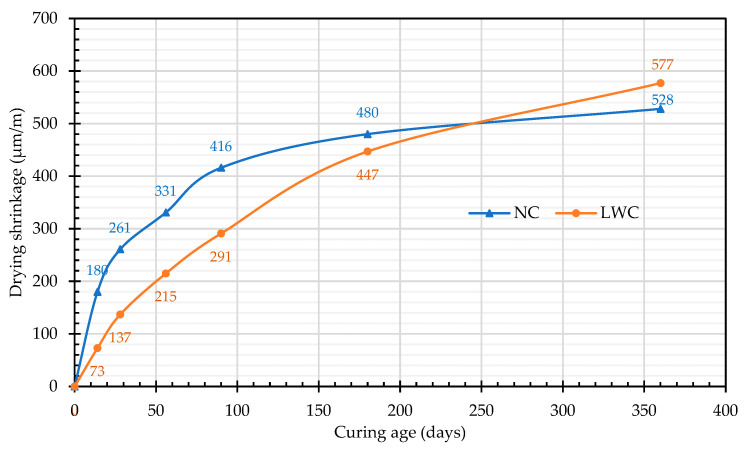
Relationship between the drying shrinkage and curing age.

In the application of LWC, one issue worthy of attention is its potential cracking under restraint conditions. To reduce the risk of cracking, LWC must have low drying shrinkage and high tensile strength. Based on this, this study conducted a drying shrinkage test. The drying shrinkage test referred to the ASTM C157 [72]. The specimen was a prism (7.5 cm × 7.5 cm × 28 cm) and was cured at the construction site. The results of the drying shrinkage test are shown in Figure 13. The drying shrinkage developed rapidly in earlier ages but slowly in later ages. For example, the 14-day drying shrinkage values of the LWC and NC were 12.7% and 34.1% of their final 360-day values, respectively. By 28 days of age, these values increased to 23.7% and 49.4%, respectively. By 90 days of age, these values increased to 50.4% and 78.8%, respectively, and continued to increase at a slower rate until 360 days of age. In addition, the initial drying shrinkage of the NC was larger than that of the LWC. This is caused by the inability to provide a sufficient moisture environment for on-site curing. However, the drying shrinkage slowed down after 90 days, as shown in Figure 13. This is due to the low permeability of normal-weight aggregates in nature, and that they are less prone to drying shrinkage than cement paste [1]. In contrast, the LWC had a smaller drying shrinkage in the early stage, but the drying shrinkage increased after 90 days of age, as shown in Figure 13. This is because the LWAs in the LWC slowly released water into the matrix, which made it more able to inhibit the early drying shrinkage, thereby making the initial drying shrinkage smaller [18]. Therefore, the drying shrinkage of the LWC at the age of 180 days was 447 microstrain (μm/m), which was smaller than that of the NC, which was 480 μm/m. However, at the age of 360 days, the dry shrinkage of the LWC was slightly higher than that of the NC due to the lower elastic modulus of the LWA. This finding was consistent with the findings of Lopez et al. [55]. Gesoğlu et al. [85] pointed out that the drying shrinkage of concrete at the age of 90 days was 640 μm/m, which was a moderately high level. Based on this, the drying shrinkage of the LWC in this study at the age of 90 days was 291 μm/m, which should be a good level. The reason for this is that the total content of cementitious materials in the LWC was not very high, only 539 kg/m^3^. However, there was a continuous supply of water in the pores of the LWAs. At the ages of 14, 28, 56, 90, 180, and 360 days, the drying shrinkage values of the LWC were 40.6%, 52.5%, 65.0%, 70.0%, 93.1%, and 109.3% of the NC, respectively. In the early stages (within six months), the drying shrinkage of the LWC was lower than that of the NC because the internal curing of the pre-soaked LWA could reduce autogenous shrinkage. But in the later period (after six months), the long-term shrinkage rate of the LWC was higher than that of the NC. This is because the LWA had lower stiffness and less restriction on the shrinkage. This is consistent with the research results in the literature [54,86].

#### 3.1.5. Results of Creep Test

**Figure 14:**
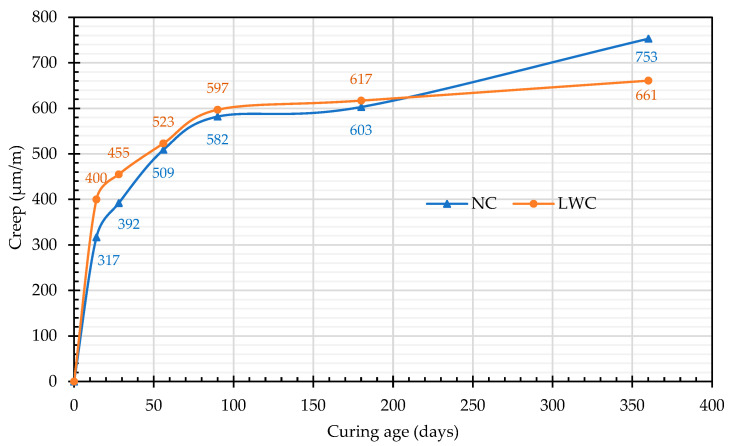
Relationship between the creep and curing age.

For accurate prestress-loss prediction, it is especially important to understand the creep behavior of concrete [47]. The results of the creep test are shown in Figure 14. The creep values of the two groups of concrete were roughly the same, but the early creep of the LWC was more evident than that of the NC, as shown in Figure 14. This is because the normal-weight aggregates generally did not exhibit appreciable creep when subjected to stress. In contrast, the porous characteristics of LWAs have a negative impact on the stiffness of LWC, which not only affects the strength but also the creep characteristics [87]. This is because the LWA present in LWC has a lower stiffness value, resulting in reduced creep resistance [16,31,88]. Overall, these experimental values were consistent with the experimental results reported in the literature [46,50,51,52,53]. At the age of 28 days, the creep of the LWC reached 455 μm/m, which was 69% of that at the age of 360 days, and it tended to be flat after 90 days. This phenomenon is presumed to be due to the lower elastic modulus of the LWC than that of the NC. Therefore, the creep under initial pressure was larger. Afterward, due to the smaller dry shrinkage, the total creep was equivalent to that of the NC. This result showed that LWC is conducive to suppressing temporal deformation because of its higher early-age-strength phenomenon and internal-curing effect. Furthermore, the matrix of the LWC had a higher strength than the matrix of the NC, which made the creep of the matrix less. This was consistent with the findings of Holste et al. [89].

**Figure 15:**
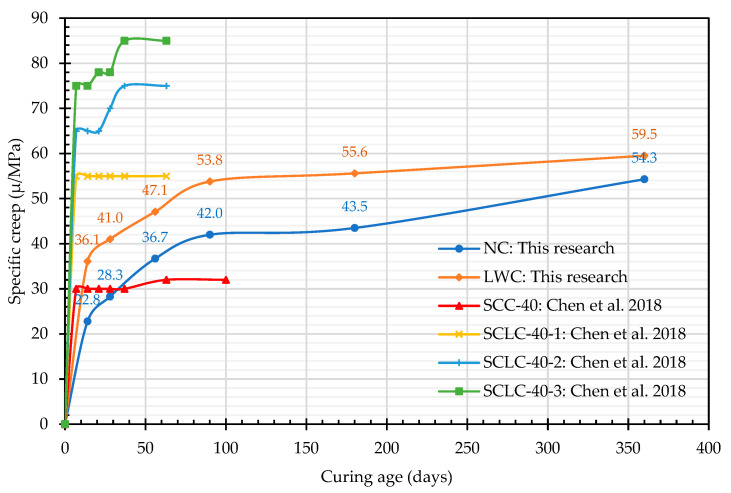
Relationship between the specific creep and curing age [16].

In order to understand the creep response of the LWC per unit of compressive stress, the value of its specific creep was also calculated, as shown in Figure 15. At the age of 28 days, the specific-creep values of the NC and LWC were 28.3 and 41.0 μ/MPa, respectively. According to Lopez et al. [59], the specific creep is 25–40 μ/MPa for high-strength NC and 75 μ/MPa for structural LWC, although it depends on the type of LWA. Accordingly, the specific creep of the LWC in this case study was significantly lower, which meant that its performance was quite excellent. This may be related to the composition of the LWC in this case study. In the composition of the LWC, silica fume, fly ash, and slag were mixed. Therefore, the cement matrix in the LWC became densified through the pozzolanic reaction of these supplementary cementitious binders, thereby reducing its specific-creep value. This is consistent with research in the literature [90,91,92,93]. In addition, the specific-creep growth trends of the two groups of concrete were particularly similar, as shown in Figure 15. However, the specific creep of the LWC was larger than that of the NC, which means that the response of the LWC to the strain deformation of the pressurized load was more evident. This is mainly because the 28-day compressive strength of the NC was 58.1 MPa and had a higher creep resistance. In comparison, the 28-day compressive strength of the LWC was 47.1 MPa and had a lower creep resistance, resulting in a greater specific creep than that of the NC. This is consistent with the study by Song et al. [94]. Nevertheless, compared with the research results of self-consolidating lightweight-aggregate concrete (SCLC) by Chen et al. [16] (Figure 15), the specific creep of the LWC in this study was significantly lower. In the study by Yen et al. [95], LWC was used to cast reduced-size prestressed beams. The specific-creep values of the 40 MPa grade LWC at 90 days and 180 days were 53 and 58.9 μ/MPa, respectively, while the specific-creep values of the 40 MPa grade NC at 90 days and 180 days were 47.6 and 52.4 μ/MPa, respectively. In this study, the 90-day and 180-day specific-creep values of the LWC were 53.8 and 55.6 μ/MPa, respectively, while the 90-day and 180-day specific-creep values of the NC were 42.0 and 43.5 μ/MPa, respectively. Accordingly, the LWC properties in this study should be excellent. This result showed that LWC can increase the compactness of its matrix through proper mix design, such as adding appropriate supplementary cementitious binders, so that it may not be worse than NC in terms of creep control.

### 3.2. Test Results of Concrete Durability

#### 3.2.1. Results of the Ultrasonic Pulse Velocity Test

This study used an ultrasonic pulse velocity (UPV) test to evaluate the compactness of the two groups of concretes. The results of the UPV test are shown in Table 9. A higher UPV indicates that the concrete has superior quality and durability, while a lower UPV indicates that the concrete is more porous. At the ages of 28 days and 180 days, the ultrasonic pulse velocities of the LWC were 4048 and 4309 m/s, respectively, while those of the NC were 4469 and 4720 m/s, respectively. The results showed that the UPV of the LWC was lower than that of the NC of the same age. However, according to the recommendation of ASTM C597 (as shown in Table 10), the compactness of the LWC was rated as of a “good” quality.

#### 3.2.2. Results for the Electrical Indication of the Concrete’s Ability to Resist the Chloride Ion Penetration Test

**Figure 16:**
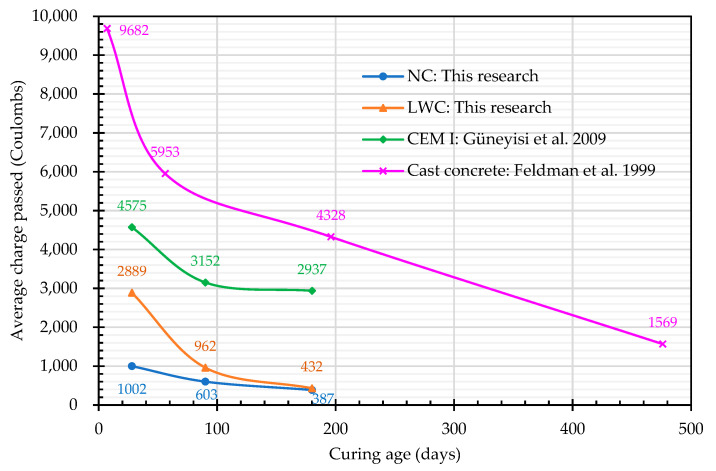
Test results of the rapid chloride permeability of the concretes [99,100].

**Figure 17:**
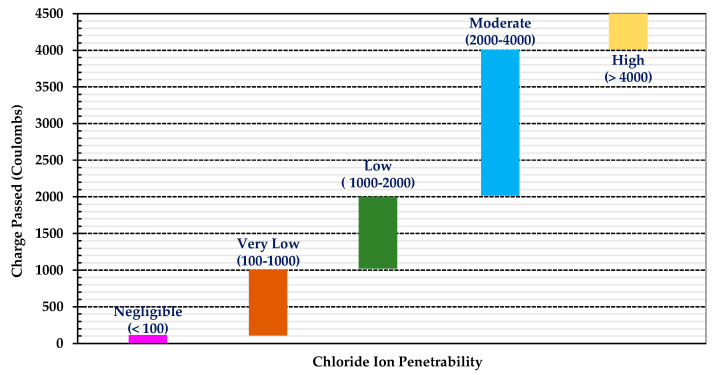
Rapid chloride permeability test ratings (ASTM C1202 [96]).

The chloride ion penetration resistance test was carried out according to CNS 14795 [74]. The sample was vacuum-saturated before testing, and the total charge passed after six hours was obtained by integrating the current over the duration. The total passed charge (coulombs) was the average of three samples, and the test results are shown in Figure 16. At the ages of 28 and 180 days, the total charges of the LWC were 2889 and 432 coulombs, respectively, while those of the NC were 1002 and 387 coulombs, respectively. According to the recommended judgment value of the chloride ion permeability (Figure 17) [96], the NC was classified as having “Very Low” or “Low” chloride permeability. At the age of 28 days, the total charge of the LWC was 2889 coulombs, and the probability of the chloride ion penetration was rated as “Moderate”. However, at the age of 180 days, the total charge of the LWC was only 432 coulombs; thus, it was classified as having “Very Low” chloride permeability. This is because the LWC contained coarse LWAs, and these porous aggregates were evenly distributed and embedded in a dense mortar matrix so that harmful substances were not able to easily penetrate into the concrete. In particular, the ITZ between the LWAs and the cement matrix was denser and could better hinder the penetration of chloride ions [97,98]. Therefore, the total passed charge of the LWC decreased significantly in the late-age stage. In addition, compared with the research results of Güneyisi et al. [99] and Feldman et al. [100], the ability of the LWC in this study to resist the chloride ion penetration was significantly better, as shown in Figure 16. In Figure 16, the CEM I concrete was made with a W/C ratio of 0.45 and a cement content of 400 kg/m^3^. Its specimens were immersed in 20 ± 2 °C water until the testing age. The mix proportions of the cast concrete were 1:2.0:3.0:0.5 (binder: fine aggregate: coarse aggregate: W/C).

#### 3.2.3. Results of the Chloride Ion Penetration Test

Chloride ion penetration is one of the most serious deterioration mechanisms affecting reinforced-concrete structures [1]. Table 11 shows the results of the chloride ion penetration test. After the LWC was soaked in a 3% sodium chloride solution for 180 days, the chloride ions increased by 1.206 and 0.495 kg/m^3^ at depths of 1.6–13 mm and 13–25 mm, respectively. However, under the same conditions, the chloride ions of the NC reached 1.490 and 1.420 kg/m^3^. The results showed that the increase in chloride ions in the LWC over a depth of 25 mm was much less than that in the NC. In other words, high-strength LWC could effectively resist chloride ion erosion. This is because although LWA has greater permeability, the protection provided by the cement matrix and the dense ITZ can enhance LWC’s resistance to chloride ion attack. This is consistent with the results in the literature [101,102,103,104]. For example, Zhang and Gjorv [103] pointed out that the permeability of high-strength LWC mainly depended on the porosity of the cement matrix rather than the porous LWA. In addition, Thomas [104] found that the use of LWA significantly reduced the electrical conductivity and chloride permeability of high-performance LWC made with a water–cement ratio of 0.30 and incorporating silica fume.

#### 3.2.4. Results of the Scanning Electron Microscope Observation

**Figure 18:**
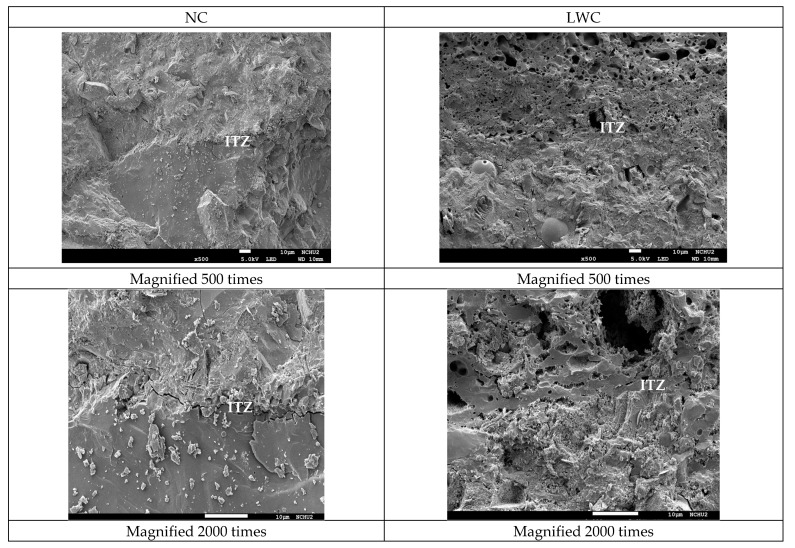
SEM micrographs of the concrete samples with a curing age of 28 days.

**Figure 19:**
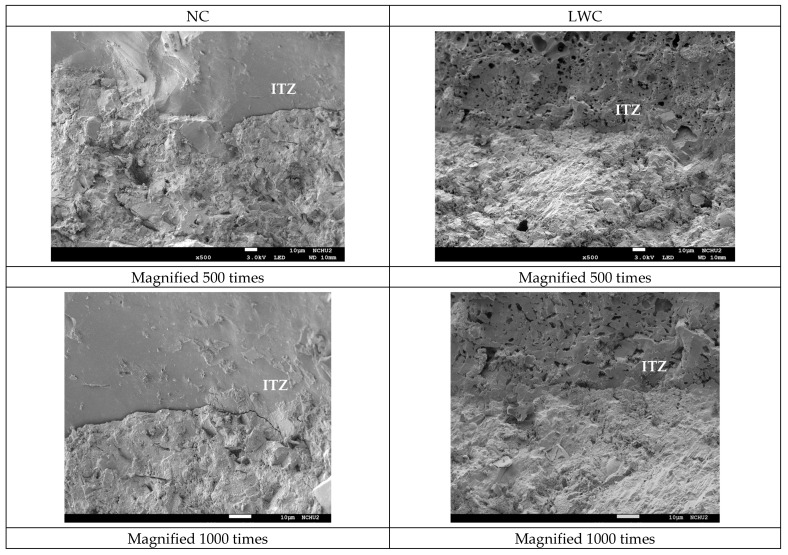
SEM micrographs of the concrete samples with a curing age of 90 days.

In concrete, the quality of the aggregate–paste interface is a key factor affecting its long-term durability. Therefore, SEM observations were conducted on the ITZs of the two groups of concretes. The SEM observation results of the two groups of concrete after curing for 28 and 90 days are shown in Figure 18 and Figure 19, respectively. It can be seen from these figures that there were obvious cracks in the ITZ of the NC. This is because, in NC, the ITZ usually has more porosity than the bulk cement matrix due to the “wall effect” [105]. In contrast, the ITZ of the LWC had no visible interfacial cracks. This is because the water absorption of the LWA in the LWC reduced the “wall effect” and the absorbed water could be used for “internal curing” during the concrete-hardening process [105]. As a result, the cement hydration increased, thereby reducing the capillary pores and their connectivity, increasing the tortuosity in the concrete, and improving the quality of the ITZ. Moreover, as mentioned before, in the LWC, the cement matrix became dense through the pozzolanic reaction of the supplementary cementitious binders. This dense microstructure protects LWA from penetration by aggressive substances [106]. Furthermore, shrinkage cracks rarely occurred in the LWC due to the release of creep constraints and the continuous supply of moisture in the pores of the LWAs. This is consistent with Newman’s research results [107]. This result indicated that the LWC could inhibit the infiltration of air, water, and chloride ions due to the strengthening of the ITZ between the LWAs and cement paste, thereby improving its durability.

### 3.3. Results of Prestress-Loss Monitoring of Prestressed Tendons

In prestressed members, prestressing is the application of compressive stress to concrete in order to relieve the tensile strain caused by a load. The various reductions in the prestressing force are called the losses in prestressing, and it is an important topic in the design of prestressed members. In this case study, the prestress value, after the prestressed box girders were prestressed and anchored for 20 min, was used as the initial prestress. On the 3rd, 7th, 14th, 28th, 56th, 90th, 180th, 360th, 540th, and 720th days, prestress-loss monitoring was carried out a total of 11 times. The monitoring data are shown in Table 12. Among them, on the 720th day, the bridge was opened to traffic. When using the magnetic-flux cable force measurement system to measure the prestress loss of the two groups of prestressed box girders, it was found that the prestress loss occurred in the initial stage. However, at 180 days, it was found that the prestress increased slightly. This is because, after the preloading of the girder, there were other subsequent works on the bridge deck, such as the laying of the AC pavement and the erection of the guardrail in New Jersey. As a result, the static load of the bridge increased, leading to a rise in the prestress.

**Figure 20:**
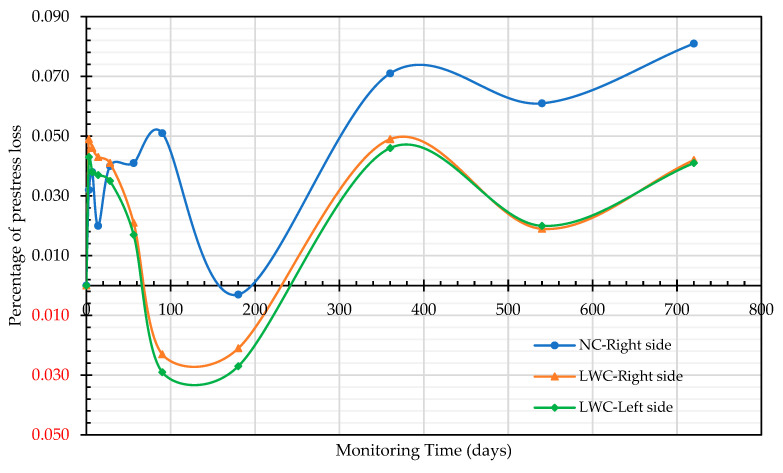
Relationship between the magnetic-flux prestress-loss percentages and monitoring times. Negative values are shown in red.

The prestress-loss rates of the two groups of prestressed box girders during the two years after prestressing are shown in Figure 20. It can be seen from Figure 20 that the prestress-loss trends of the two groups of prestressed box girders are similar. The largest prestress loss of the NC prestressed box girder occurred on the 720th day, and the prestress loss of magnetic flux reached 8.1%. In contrast, the largest prestress loss of the LWC prestressed box girder occurred on the 360th day after prestressing, and the magnetic-flux prestress-loss-monitoring values on both sides were 4.6% and 4.9%, respectively. The test results of Chen et al. [16] showed that, after 180 days of prestressing, the prestress loss was about 5.35–6.83% for the full-size self-consolidating lightweight-aggregate-concrete (SCLC) members, which was smaller than that for the conventional self-consolidating-concrete (SCC) members (about 8.19%–9.06% loss). Moreover, in the study by Mohebbi and Graybeal [108], seven full-scale prestressed ultra-high-performance-concrete (UHPC) girders with 17.8 mm diameter strands and different geometries were constructed using two commercially available UHPC products (U-H and U-J). Their test results showed that the total prestress loss of all the girders varied between 12.4% and 22.5%. Compared with the literature [16,108], the prestress losses of the two groups of prestressed box girders were not high. In particular, the prestress loss of the LWC prestressed box girder was smaller than that of the NC prestressed box girder.

### 3.4. Summary of Test Results

At present, the development of synthetic LWAs mainly uses industrial waste or municipal solid waste as raw materials. For example, slag, fly ash, reservoir sediment, waste TFT-LCD glass powder, paper sludge, tile-grinding sludge, water purification sludge, textile sludge, and other renewable resources are used to produce LWAs [5,6,7,8,9,10,11,12,13,14,15]. Many studies have shown the great potential of artificial aggregates made from industrial by-products or solid waste as substitutes for natural coarse aggregates [109]. However, the behavior of LWC can vary greatly depending on several factors, such as the type of LWA, the curing conditions, the matrix quality, the moisture content, and the penetration mechanism [110]. In other words, most studies focus on types of LWAs, binder types, or environmental exposure categories that are only valid for the specific case studied [111]. This may plausibly explain the apparently contradictory results reported in the literature.

This case study evaluated the basic mechanical properties and durability of synthetic lightweight-aggregate concrete made from reservoir sediments. It can be seen from the durability test results that the ultrasonic pulse velocity of the LWC used in this case was lower than that of the NC at different ages. However, the compactness of the LWC was estimated to be within the category of good quality based on regulatory standards. In addition, the chloride ion penetration test results showed that the probability of the chloride ion permeability of the LWC was very low at the age of 90 days. The test results using the CNS 14702 acid-soluble chloride ion content test method also showed that the increase in chloride ions inside the LWC over a depth of 25 mm was much less than that in the NC. This test result proved that the LWC used in this case study could effectively resist erosion by chloride ions and external moisture. This is consistent with the research results in the literature [112,113,114]. Moreover, it can be seen from the SEM images that there were obvious interfacial cracks in the ITZ between the aggregate and cement paste in the NC, while there were no visible interfacial cracks in the ITZ between the aggregate and cement paste in the LWC. This observation once again illustrated that the LWC could inhibit the infiltration of air, water, and chloride ions due to the strengthening of the ITZ between the LWAs and cement paste, thus improving the durability of the concrete. From the prestress-loss monitoring, it can be found that the prestress-loss-monitoring value of the LWC box girder was lower than 4.9% in two years, which was far lower than the design value of 12.0% and the monitoring value of 8.1% in the NC.

The above test results illustrated that the LWC with a compressive strength of 40 MPa was successfully manufactured from synthetic LWAs made from reservoir sediments, and its engineering properties complied with ACI 213R-14 [17]. This indicated that an effective strategy to improve the durability of LWC is to design a dense microstructure of the cement matrix so that its engineering performance is not inferior to that of NC [106]. In particular, the higher early-age-strength phenomenon and internal-curing effect of LWC are beneficial to suppressing temporal deformation, thereby reducing prestress loss. Furthermore, LWCs have a small self-weight, which can reduce the bending moment of the prestressed beam and the bridge deck, thereby reducing the section of the component that is used to achieve a larger span.

## 4. Conclusions

This case study rigorously compared the durability and prestress-loss differences in NC and LWC prestressed box girders constructed simultaneously in the same area through comprehensive experimental methods. The test results verify the superiority of using synthetic LWA made from reservoir sediments in prestressed bridges. Under standard-curing conditions, the 28-day compressive strength of the LWC was 47.1 MPa, meeting the requirements of ACI 213R-14. Due to the internal-curing effect and lower elastic modulus of the LWA, the total dry shrinkage and total creep of the LWC were comparable to those of the NC. Furthermore, LWC can inhibit the infiltration of air, water, and chloride ions due to the reduced deformation and improved microstructure in its ITZ, thereby improving its durability. Notably, the magnetic-flux prestress loss of the NC prestressed box girder reached 8.1%. In contrast, the monitored values of the magnetic-flux prestress loss on both sides of the LWC prestressed box girder were 4.6% and 4.9%, respectively. This verified that, under the same environmental conditions, the use of LWC produced less of a prestress loss than the use of NC.

## Figures and Tables

**Table 1:** Chemical compositions of the cementitious materials.

Chemical Composition (%)	Cement	Slag	Fly Ash	Silica Fume
Silicon dioxide, SiO_2_	20.49	33.46	55.90	92.40
Aluminum oxide, Al_2_O_3_	6.57	13.70	29.30	-
Iron oxide, Fe_2_O_3_	3.27	0.42	4.05	-
Calcium oxide, CaO	62.40	42.69	-	-
Magnesium oxide, MgO	1.91	6.21	-	-
Sulfur trioxide, SO_3_	2.20	1.48	0.42	-
Potassium oxide, K_2_O	-	0.35	-	-
Free calcium oxide, f-CaO	1.03	-	-	-
Titanium dioxide, TiO_2_	-	0.46	-	-
Phosphorus Pentoxide, P_2_O_5_	-	0.04	-	-
Manganese oxide, MnO	-	0.39	-	-
Loss on ignition, LOI	1.57	0.27	3.64	1.50
Tricalcium silicate, C_3_S	39.27	-	-	-
Dicalcium silicate, C_2_S	29.11	-	-	-
Tricalcium aluminate, C_3_A	11.75	-	-	-
Tetracalcium aluminoferrite, C_4_AF	9.96	-	-	-
Calcium sulfate dihydrate, CSH_2_	4.73	-	-	-

**Table 2:** Basic properties of lightweight aggregates.

Item		Test Value	Test Method
Bulk specific gravity		1.32	ASTM C127 [62]
Crushing strength		4.9 MPa	CNS 14779 [63]
Los Angeles abrasion value of aggregate		31.20%	CNS 490 [64]
Soundness of aggregate via use of sodium sulfate or magnesium sulfate		0.11%	CNS 1167 [65]
Water absorption rate	24 h	10%	ASTM C127 [62]
	48 h	12%	
Dry unit weight		835 kg/m^3^	CNS 3691 [66]

**Table 3:** Mix proportions of the concretes (kg/m^3^).

Concrete Group	W/B	W	C	SL	FA	SF	LWA	CA	FA	SP
Control group	0.36	163	364	90	0	0	0	971	774	5.0
Experimental group	0.39	210	296	162	54	27	465	0	615	4.85

Notes—W/B: water–binder ratio; W: water; C: cement; SL: slag; FA: fly ash; SF: silica fume; LWA: lightweight aggregate; CA: coarse aggregate; FA: fine aggregate; SP: superplasticizer.

**Table 4:** The test items, specimen ages, and test specifications of the basic properties and time-dependent deformation of the concretes.

Test Items	Specimen Ages (Day)	Specimen Size	Test Specifications
Air-dry unit weight	90	Cylinders (15 cm diameter × 30 cm length)	ASTM C567 [67]
Compressive strength	3, 7, 14, 21, 28, 56, 90, 180	Cylinders (15 cm diameter × 30 cm length)	ASTM C39 [68]
Flexural strength	28, 56, 90, 180	Prisms (15 cm × 15 cm × 53 cm)	ASTM C78 [69]
Splitting tensile strength	28	Cylinders (15 cm diameter × 30 cm length)	ASTM C496 [70]
Elastic modulus	28, 56, 90, 180	Cylinders (15 cm diameter × 30 cm length)	ASTM C469 [71]
Drying shrinkage	0, 14, 28, 56, 90, 180, 360	Prisms (7.5 cm × 7.5 cm × 28 cm)	ASTM C157 [72]
Creep	0, 14, 28, 56, 90, 180, 360	Cylinders (15 cm diameter × 30 cm length)	ASTM C512 [73]

**Table 5:** The test items, specimen ages, and test specifications of concrete durability.

Test Items	Specimen Ages (Day)	Specimen Size	Test Specifications
Ultrasonic pulse velocity test	28, 90, 180	Cylinders (10 cm diameter × 20 cm length)	ASTM C597 [77]
Concrete’s ability to resist chloride ion penetration test	28, 90, 180	Cylinders (10 cm diameter × 20 cm length)	CNS 14795 [74]
Chloride ion penetration test	28, 90, 180	Cylinders (10 cm diameter × 20 cm length)	ASTM C1543 [75] and CNS 15649 [76]
Scanning electron microscope observation	28, 90, 180	Fragments after concrete compression test	ACI 213R-14 [17]

**Table 6:** Test results of the air-dried unit weights of the concretes.

Group	Curing Method	Air-Dried Unit Weight (kg/m^3^)
		90 Days
NC	Standard curing	2391
	On-site curing	2333
LWC	Standard curing	1817
	On-site curing	1820

**Table 7:** Test results of the compressive strengths of the concretes.

Group	Curing Method	Curing Age (Day)							
		3	7	14	21	28	56	90	180
NC	Standard curing	25.6	35.6	50.2	57	58.8	68.9	74.5	75.1
	On-site curing	-	-	-	-	61.6	65.4	64.7	67.1
LWC	Standard curing	24.24	33.6	39.9	45.6	47.1	50.6	52.4	56.1
	On-site curing	-	-	-	-	51.0	51.6	52.4	53.3

**Table 8:** Test results of the splitting tensile strengths of the concretes.

Group	Curing Method	Splitting Tensile Strength (MPa)
		28 Days
NC	Standard curing	4.6
	On-site curing	4.5
LWC	Standard curing	3.0
	On-site curing	2.3

**Table 9:** Test results of the ultrasonic pulse velocities of the concretes.

Group	Curing Age (Day)	Ultrasonic Pulse Velocity (m/s)	Average UPV (m/s)
NC	28	4470.4, 4452.8, 4483.2	4469
	90	4608.8, 4604.3, 4682.4	4632
	180	4724.1, 4733.6, 4701.8	4720
LWC	28	4067.5, 4053.7, 4022.4	4048
	90	4252.7, 4188.6, 4231.6	4224
	180	4332.2, 4308.2, 285.7	4309

**Table 10:** Ultrasonic pulse velocities of the concrete and quality judgments.

UPV Range (m/s)	Concrete Quality
More than 4500	Excellent
From 3600 to 4500	Good
From 3000 to 3600	Questionable
From 2100 to 3000	Poor
From 1800 to 2100	Very poor

**Table 11:** Test results of the chloride ion penetration test.

Group	Curing Age (Day)	Increase in Chloride Ion Content (kg/m^3^)			
		Sampling Position: 1.6–13 mm	Difference from Comparison Sample	Sampling Position: 13–25 mm	Difference from Comparison Sample
NC	0	0.030	0.000	0.033	0.000
	28	0.524	0.494	0.293	0.260
	90	1.270	1.240	1.210	1.177
	180	1.521	1.490	1.453	1.420
LWC	0	0.096	0.000	0.076	0.000
	28	0.680	0.584	0.097	0.021
	90	0.918	0.822	0.307	0.231
	180	1.302	1.206	0.571	0.495

**Table 12:** Prestress values in the magnetic-flux monitoring of prestressed box girder (unit: ton).

Installation Location		Monitoring Time (Day)										
		0	3	7	14	28	56	90	180	360	540	720
NC Right side		376	364	362	369	361	361	357	377	350	353	346
LWC	Right side	265	252	253	254	254	260	271	271	252	260	254
	Left side	268	257	258	258	259	264	276	276	256	263	257

## Data Availability

The data presented in this study are available upon request from the corresponding authors.
